# High Glucose Impairs Insulin Signaling in the Glomerulus: An *In Vitro* and *Ex Vivo* Approach

**DOI:** 10.1371/journal.pone.0158873

**Published:** 2016-07-19

**Authors:** Elias N. Katsoulieris, Garyfalia I. Drossopoulou, Eleni S. Kotsopoulou, Dimitrios V. Vlahakos, Elias A. Lianos, Effie C. Tsilibary

**Affiliations:** 1 Institute of Biosciences and Applications, National Center for Scientific Research ‘Demokritos’, Athens, Greece; 2 2nd Department of Propaedeutic Medicine, Attikon University Hospital, Athens, Greece; 3 Department of Pathology, National and Kapodistrian University of Athens, Medical School, Athens, Greece; Virgen Macarena University Hospital, School of Medicine, University of Seville, SPAIN

## Abstract

**Objective:**

Chronic hyperglycaemia, as seen in type II diabetes, results in both morphological and functional impairments of podocytes in the kidney. We investigated the effects of high glucose (HG) on the insulin signaling pathway, focusing on cell survival and apoptotic markers, in immortalized human glomerular cells (HGEC; podocytes) and isolated glomeruli from healthy rats.

**Methods and Findings:**

HGEC and isolated glomeruli were cultured for various time intervals under HG concentrations in the presence or absence of insulin. Our findings indicated that exposure of HGEC to HG led to downregulation of all insulin signaling markers tested (IR, p-IR, IRS-1, p-Akt, p-Fox01,03), as well as to increased sensitivity to apoptosis (as seen by increased PARP cleavage, Casp3 activation and DNA fragmentation). Short insulin pulse caused upregulation of insulin signaling markers (IR, p-IR, p-Akt, p-Fox01,03) in a greater extent in normoglycaemic cells compared to hyperglycaemic cells and for the case of p-Akt, in a PI3K-dependent manner. IRS-1 phosphorylation of HG-treated podocytes was negatively regulated, favoring serine versus tyrosine residues. Prolonged insulin treatment caused a significant decrease of IR levels, while alterations in glucose concentrations for various time intervals demonstrated changes of IR, p-IR and p-Akt levels, suggesting that the IR signaling pathway is regulated by glucose levels. Finally, HG exerted similar effects in isolated glomeruli.

**Conclusions:**

These results suggest that HG compromises the insulin signaling pathway in the glomerulus, promoting a proapoptotic environment, with a possible critical step for this malfunction lying at the level of IRS-1 phosphorylation; thus we herein demonstrate glomerular insulin signaling as another target for investigation for the prevention and/ or treatment of diabetic nephropathy.

## Introduction

Diabetes remains the most common cause of end-stage renal failure in the United States and worldwide with between 20 and 40% of those with diabetes mellitus developing some degree of nephropathy [1,2]. At the onset of diabetes, the kidney becomes enlarged and the GFR increases [3] without overt clinical signs and symptoms. The earliest appearing symptom is the presence of albumin in the urine (albuminuria) which can develop into nephrotic-range proteinuria, accompanied by morphological changes, such as glomerular hypertrophy, glomerular basement membrane thickening and extracellular matrix (ECM) expansion. Decreased GFR, glomerulosclerosis, glomerular capillary dysfunction and tubulointerstitial degeneration constitute the adverse outcomes of advanced diabetic nephropathy. Current treatments aim only to slow the progression of the disease, and these include strict blood glucose and pressure control and inhibition of the renin-angiotensin system [4,5].

The glomerular filtration barrier is composed of glomerular endothelial cells and podocytes, the latter being pivotal for maintaining the integrity of the barrier function. Podocyte loss is evident in early diabetic nephropathy in humans and largely determines how rapidly the disease will progress [6–9]. Various mechanisms are involved in high glucose (HG)-induced podocytic injury and apoptosis and include increased renin-angiotensin-aldosterone signaling [10,11], oxidative stress [12], advanced glycation end products (AGEs) formation [13,14], and PKC activation [15]. In addition, nephrin expression, a protein which contributes to podocyte survival by inhibiting proapoptotic signaling [16], is reduced in diabetes [17] and highly secreted in urine [18], an indication of podocyte injury.

Both type 1 and type 2 diabetes are associated with peripheral insulin resistance [19,20]. Insulin resistance is characterized by inability of insulin to exert its signaling effects, mainly due to dysfunction at the level of IRS1 and IRS2 activation and PI3K recruitment to the plasma membrane [21]. In the kidneys, insulin resistance is correlated with microalbuminuria in type 2 diabetes patients [22] and type 2 diabetic rat models [23]. In the glomerular barrier, only the podocyte is insulin-responsive [24]; glomerular insulin signaling is critical for glomerular filtration barrier integrity and normal kidney function [25]. In addition, it has been reported that there is reduced expression of insulin receptors in the kidneys of insulin-resistant rats [26] and that podocytes from db/db diabetic mice have diminished insulin responses [27]. Beside the traditional target organs of insulin action (liver and skeletal muscle), the cardiovascular system and the kidney have been also recognized to be insulin targets. It is possible that disruption of normal insulin signaling (hyperinsulinemia, insulin resistence or absolute insulin deficiency) may play a significant role in the pathogenesis of diabetic complications. Furthermore, renal disease similar to diabetic nephropathy can be observed in patients with a genetic mutation of the insulin receptor, which would suggest that disruption of normal insulin signaling is a part of the disease process in diabetic nephropathy [Spectrum of renal diseases associated with extreme forms of insulin resistance [28]. It is interesting to note that insulin can influence glomerular permeability to albumin in patients with type 2 diabetes but not in healthy subjects, suggesting that disruption of the insulin signaling cascade may be sufficient for insulin to result in microalbuminuria in type 2 diabetes [29]. Hence, elucidation of the mechanisms involved in the downregulation of insulin signaling in podocytes will contribute to successfully interfering with this impairment. We provide evidence herein that high glucose itself impairs insulin signaling in an immortalized human podocyte cell line, as well as in isolated rat glomeruli, which is leading to apoptotic cell death.

## Methods

### Culture of human glomerular epithelial cells (HGEC)

HGEC (kind gift of Dr. Delarue) [30] were cultured as previously described [31]. More specifically, T-SV40-immortalized human glomerular epithelial cells (HGEC) were cultured at 37°C in an environment of 5% CO2 in media composed of DMEM/Ham’s F-12 containing 1% FCS, 15 mM HEPES, 2 mM glutamine, ITS (5 mg/ml insulin, 5 ng/ml sodium selenite, 5 mg/ml transferrin), 50 nM dexamethasone, 100 U/ml penicillin, 100 mg/ml streptomycin, 25 mg/ml amphotericin and 5 mM D-glucose (termed as NG-treated HGEC). We have also used HGEC cultured in 25 mM D-glucose for a minimum of 6 months before performing experiments (termed as chronic treatment of HGEC with HG). The media were replaced every second day. At subconfluency (≈ 80% confluency) HGEC were subcultured using a combination of 0.05% (w/v) trypsin and 0.03% (w/v) ethylenediaminetetraacetic acid (EDTA, versene) in 6- or 24- well plates (coverslips were used where required).

### Incubation with insulin and treating solutions

For insulin signaling experiments, and prior to lysis, cultured HGEC and glomeruli were incubated in Krebs buffer {118.5 mmol/L NaCl, 2.54 mmol/L CaCl_2_, 1.19 mmol/L KH_2_PO_4_, 25 mM NaHCO_3_, 1.19 mmol/L MgSO_4_7H_2_O, 10 mmol/L HEPES, 0.1% (w/v) BSA, pH 7.4} for 2 h; fresh buffer was applied every 30 min. In experiments in which an ‘insulin pulse’ was performed, cells or glomeruli were incubated with 100 nmol/L short-acting commercially available insulin (Actrapid) for 15 minutes.

For prolonged insulin treatment experiment, long-acting commercially available insulin (Levemir) was used, which produced similar effects to Actrapid at a concentration of 300 nmol/L. Regarding these series of experiments, HGEC were incubated with 300 nmol/L insulin (Levemir) for 24h, directly applied to the culture medium. In order to verify phosphoinositide 3-kinase (PI3K)-dependent phosphorylation, the PI3K inhibitor wortmannin (Sigma) was used in the relevant series of experiments and applied directly to the cell culture media at a concentration of 200nmol/L for 24 h prior to insulin treatment. Similarly, the apoptotic inducers dithiothreitol (DTT) and staurosporine (SP) were added to the tissue culture media directly for 24 or 48h.

### Western blotting

Total protein was extracted from HGEC or isolated glomeruli using the modified protein extraction buffer (RIPA) {150 mmol/L NaCl, 50 mmol/L Tris-HCl (pH 7.4), 1 mmol/L Na_2_EDTA, 1% (v/v) Triton-X 100, 0,25% (w/v) sodium deoxycholate and 0.1% (w/v) sodium dodecyl sulphate (SDS)} containing protease inhibitor cocktail (Roche Diagnostics, Athens, Greece) [32]. Following centrifugation at 10,000 g for 30 min at 4°C, supernatants were collected and assayed for protein content using the Bradford assay [33]. 30–90 μg of protein was loaded into each lane of a 7,5% polyacrylamide gel, in the presence of SDS, and proteins were allowed to separate at 120 V for 90 min. Transfer of proteins to nitrocellulose membranes (Amersham) was performed at 4°C at 100 V for 80 min. The membranes were then blocked using 5% (w/v) powdered milk for 1 h and incubated with the primary antibodies overnight at 4°C. All antibodies were obtained from Cell Signaling Technology (Bioline Scientific, Athens, Greece), unless otherwise stated. The primary antibodies used and final concentrations (which were based on manufacturer's recommendations) were: rabbit polyclonal anti-PARP, 1:1000 dilution; rabbit monoclonal anti-PI3K (Merck Millipore) 1:1000 dilution; rabbit polyclonal anti-p-Fox01 [pT^24^]/03 [pT^32^] (Invitrogen), 1:1000 dilution; mouse monoclonal anti-β-tubulin (Sigma-Aldrich), 1:500 dilution; rabbit monoclonal anti-p-Akt (Ser473), 1:2000 dilution; rabbit polyclonal anti-cleaved Caspase-3, 1:500 dilution; rabbit polyclonal anti-IRS1, 1:250 dilution; rabbit polyclonal anti-p-IRS1(Ser636) (Santa Cruz Biotechnology), 1:200 dilution; rabbit polyclonal anti-p-IRS1(Tyr465) (Santa Cruz Biotechnology), 1:200 dilution; rabbit monoclonal anti-p-IGF-IRβ, 1:1000 dilution; rabbit polyclonal anti-IRβ (Santa Cruz Biotechnology), 1:200 dilution; rabbit polyclonal anti-Akt, 1:1000 dilution. Immunodetection was performed at room temperature for 1 h using an appropriate secondary antibody diluted 1:5000 in Tween-Tris buffered saline containing 1 or 5% (w/v) milk or BSA. Visualization of protein bands was performed using an ECL Plus chemiluminescent detection system (Amersham,). Densitometric analysis was performed using Image J and the results were normalized using β-tubulin.

### Glomeruli isolation and culture

Male Wistar rats weighing 200-250g were used for the *ex vivo* study (obtained from Charles River Laboratories). Rats were maintained with free access to water and rat chow. Experimental protocols were approved by the Institutional Animal Care and all animal experimentations were carried out in agreement with the ethical recommendations of the European Communities Council Directive of 22 September 2010 (2010/63/EU), animal welfare assurance number: protocol nbr: 6464. All procedures were performed under phenobarbital anaesthesia. Glomeruli isolation was carried out as described by Sharma and colleagues [34]. Briefly, a high mid line incision on the abdomen exposed the abdominal cavity and total bilateral nephrectomy was performed to anesthetized animals. The kidney capsules were removed and glomeruli were isolated following consecutive passage of the mashed cortexes through screens of 80- and 200-mesh size. Glomeruli were recovered from atop the 200 mesh screen into DMEM supplemented with 10% (v/v) FBS. These were then transferred to 6-well culture plates, incubated for 4 days with the same culture media used for HGEC culture, containing 5- or 25- mmol/L glucose.

### DNA isolation and gel electrophoresis (DNA laddering)

Total genomic DNA was isolated from HGEC and allowed to separate into fragments via agarose gel electrophoresis, as previously described [35]. DNA was extracted from both floating (detached) and still adherent cells following exposure to apoptotic stimuli. Cells were lysed in lysis buffer {50 mmol/L Tris-HCl pH 8.0, 100 mmol/L EDTA, 100 mmol/L NaCl, 1% (v/v) SDS} containing 1 mg/mL Proteinase K (Roche Diagnostics, Athens, Greece) and 1 mg/mL RNAse A (Sigma-Aldrich, Life Sciences Chemilab, Athens, Greece) for 2 h at 55°C and then an equal volume of phenol/chloroform/isoamylalcohol mixture was added. Following centrifugation at 12,000 rpm for 10 min at room temperature, the upper phase was collected and mixed with an equal volume of isopropanol. The mixture was centrifuged at 12,000 rpm at 4°C for 10 minutes and the pellet (DNA) was ethanol (75% v/v)-washed, air-dried and re-dissolved in mq H_2_O. Following measurement of DNA concentration at Nanodrop, DNA samples were loaded onto a 2% agarose gel containing 0.1 mg/mL ethidium bromide and electrophoresis occurred in TBE buffer at 80 V for 2h. The resulting fragments were visualized under UV light and photographed.

### In situ cell death detection—TUNEL staining

HGEC grown on glass coverslips were fixed with 4% (w/v) paraformaldehyde in PBS pH 7.4, for 1 hour at room temperature, permeabilized for 2 min on ice with 0.1% TritonX-100 in 0.1% sodium citrate and finally incubated with 50 μl TUNEL reaction mixture (TdT enzyme and fluorochrome labeling solution) for 1 hour at 37°C in the dark [In Situ Cell Death Detection Kit, TMR red (Roche Diagnostics, Mannheim, Germany)]. Finally, the cells were washed with PBS, and incubated with DAPI (1 μg/ml) in PBS for 5 min, before mounting with Dako Fluorescent Mounting Medium (Cat. No: S3023, Dako). Specimens were examined with a confocal laser-scanning microscope (TCS SP5 Confocal System, Leica). Images were obtained and processed with Adobe Photoshop CS4 version 11.0, software. Quantification of the percentage of cells undergoing apoptosis was performed in digital images.

### Statistical analysis

Results are expressed as mean ± standard error of the mean (SEM) for n independent observations as indicated. Statistical differences between mean values of groups were determined using either non-paired t-tests for comparison of two means or one way analysis of variance (ANOVA) followed by Dunnett's post-significance test for comparison of multiple means, with commercially available software (Graphpad Prism, version 4.0, Graphpad Software, San Diego, CA, USA). The level of significance was set at P<0.05.

## Results

### High glucose results in downregulation of insulin signaling in HGEC

Modulation of the insulin signaling pathway markers in response to HG was studied in the presence and absence of short term (15 min) insulin treatment, termed as ‘insulin pulse’ which was anticipated to stimulate activation of the IR by tyrosine phosphorylation and enhance the downstream signaling response.

**Fig 1:**
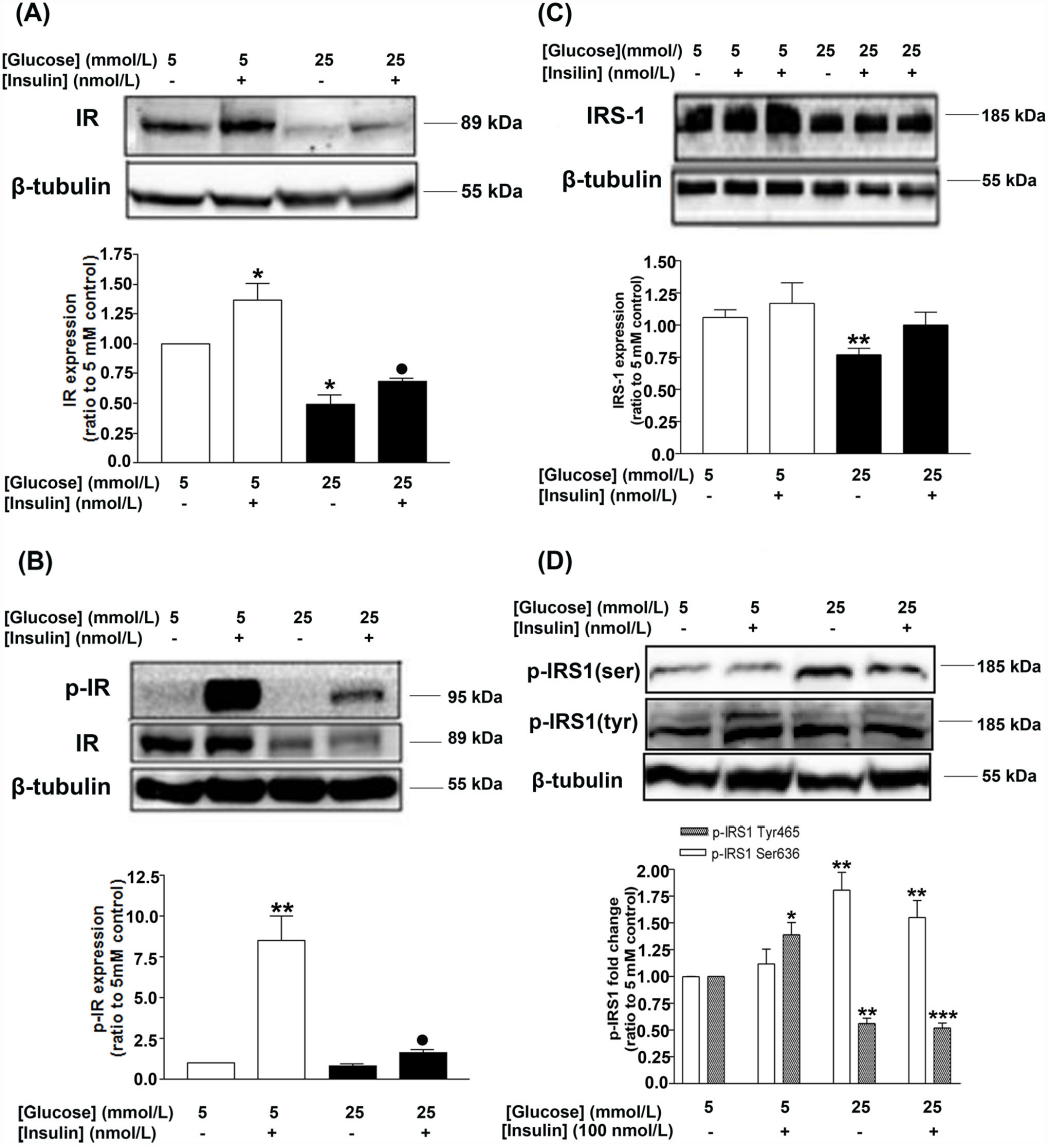
Effects of chronic HG on the insulin signaling pathway of HGEC in the presence and absence of insulin pulse (15 min treatment). HG results in downregulation of the expression (A; Western blot and densitometric analysis) and activation levels of IR (B; Western blot and densitometric analyses) and the expression levels of IRS-1 (C; Western blot and densitometric analysis). HG results in downregulation of tyrosine residue phosphorylation and upregulation of serine residue phosphorylation in IRS-1 (D; Western blot and densitometric analysis). Data represent mean±SEM, n = 3–5, *P<0.05, **P<0.01 vs. corresponding control (5 mmol/L glucose) and •P<0.05 vs. 25 mmol/L glucose control.

Chronic treatment of HGEC with HG caused downregulation of IR basal levels, while an insulin pulse triggered significant increases of IR levels in both HG- and NG-treated cells, to a similar extent (Fig 1A). Phosphorylation of IR levels following insulin treatment in the HG-treated group of cells was greatly reduced when compared to control NG-treated cells (Fig 1B), suggesting that the activation response of IR to insulin is compromised under chronic HG conditions.

Moreover, IRS-1 basal levels in HG-treated cells were reduced compared to cells which were grown in NG (Fig 1C). Following insulin treatment, IRS-1 levels of HG-treated cells were not significantly different compared to cells grown in NG (Fig 1C). This finding suggested that insulin treatment could overcome the inhibitory effect of hyperglycemia (Fig 1C). Concomitantly, however, phosphorylation of IRS-1 at Ser636 was increased while phosphorylation at Tyr465 was reduced (Fig 1D). Short term insulin treatment had no effect on IRS-1 Ser636 phosphorylation; however, it enhanced Tyr465 phosphorylation levels in NG-treated, but not in HG-treated cells (Fig 1D).

**Fig 2:**
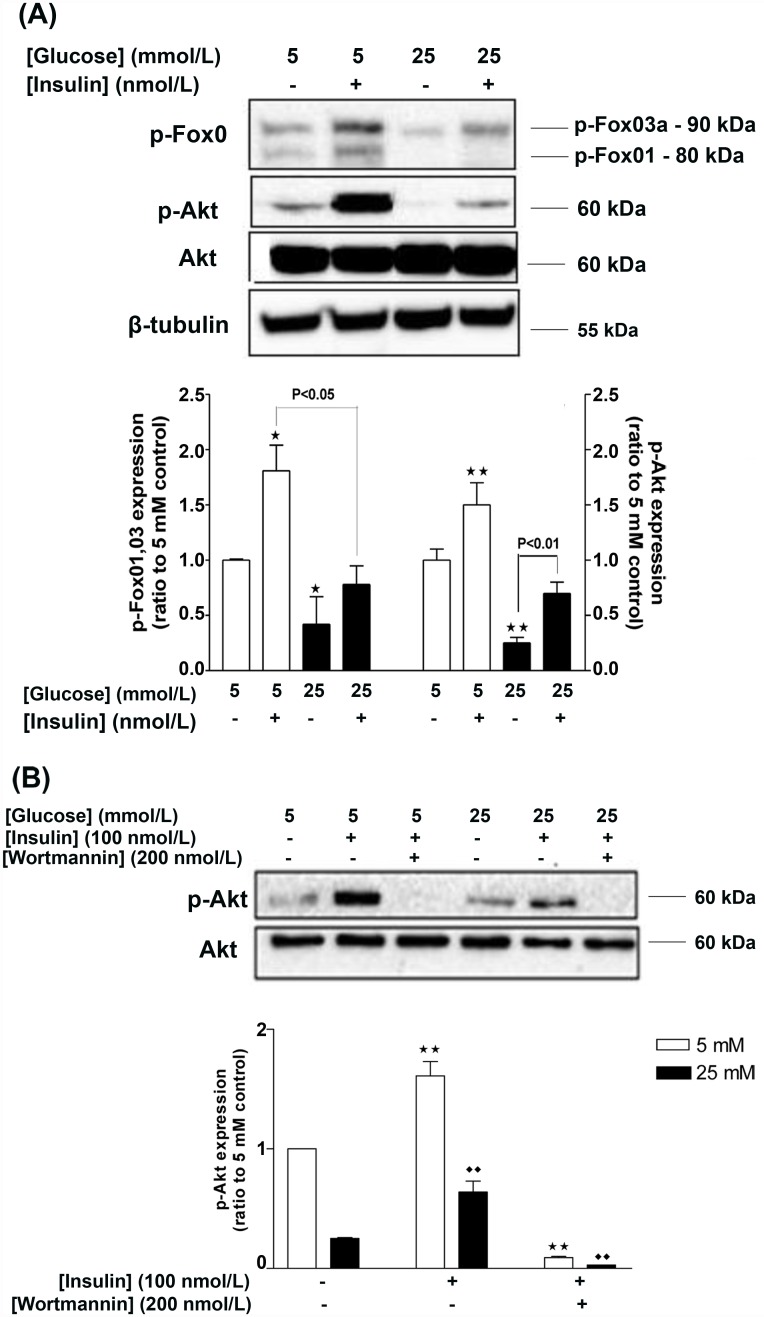
Effects of chronic HG on the downstream insulin signaling pathway of HGEC in the presence and absence of insulin pulse (15 min treatment). HG results in downregulation of the phosphorylation levels of Akt and Fox01,03a (A; Western blot and densitometric analysis). Insulin action on p-Akt and p-Fox01,03a is PI3K-dependent, as pretreatment of HGEC for 24 h with the PI3K inhibitor wortmannin, abolished the stimulatory effects of insulin (15 min treatment) on p-Akt (B; Western blot and densitometric analysis). Data represent mean±SEM, n = 3, **P<0.01 vs. control (5 mmol/L glucose) and ^••^P<0.01 vs. control (25 mmol/L glucose).

The downstream insulin signaling effectors, Akt and the transcription factor Fox01,03a exhibited decreased phosphorylation in response to HG, which was partially reversed by insulin pulse as far as p-Akt was concerned (Fig 2A). Insulin pulse increased phosphorylation of Fox01,03a in NG-treated cells, but not in HG-treated cells (Fig 2A). Moreover, basal Akt phosphorylation was reduced by approximately 50% in HG-treated cells, whereas short insulin treatment augmented p-Akt levels in both groups to a similar extent (Fig 2A). Pretreatment of HGEC with the PI3K inhibitor wortmannin (200 nmol/L) [36] for 24 h abolished both basal and insulin-mediated increases of Akt phosphorylation in both NG- and HG-treated groups, indicating that phosphorylation of Akt is PI3K-dependent (Fig 2B).

### Effects of glucose concentration alterations and prolonged insulin treatment in HGEC

**Fig 3:**
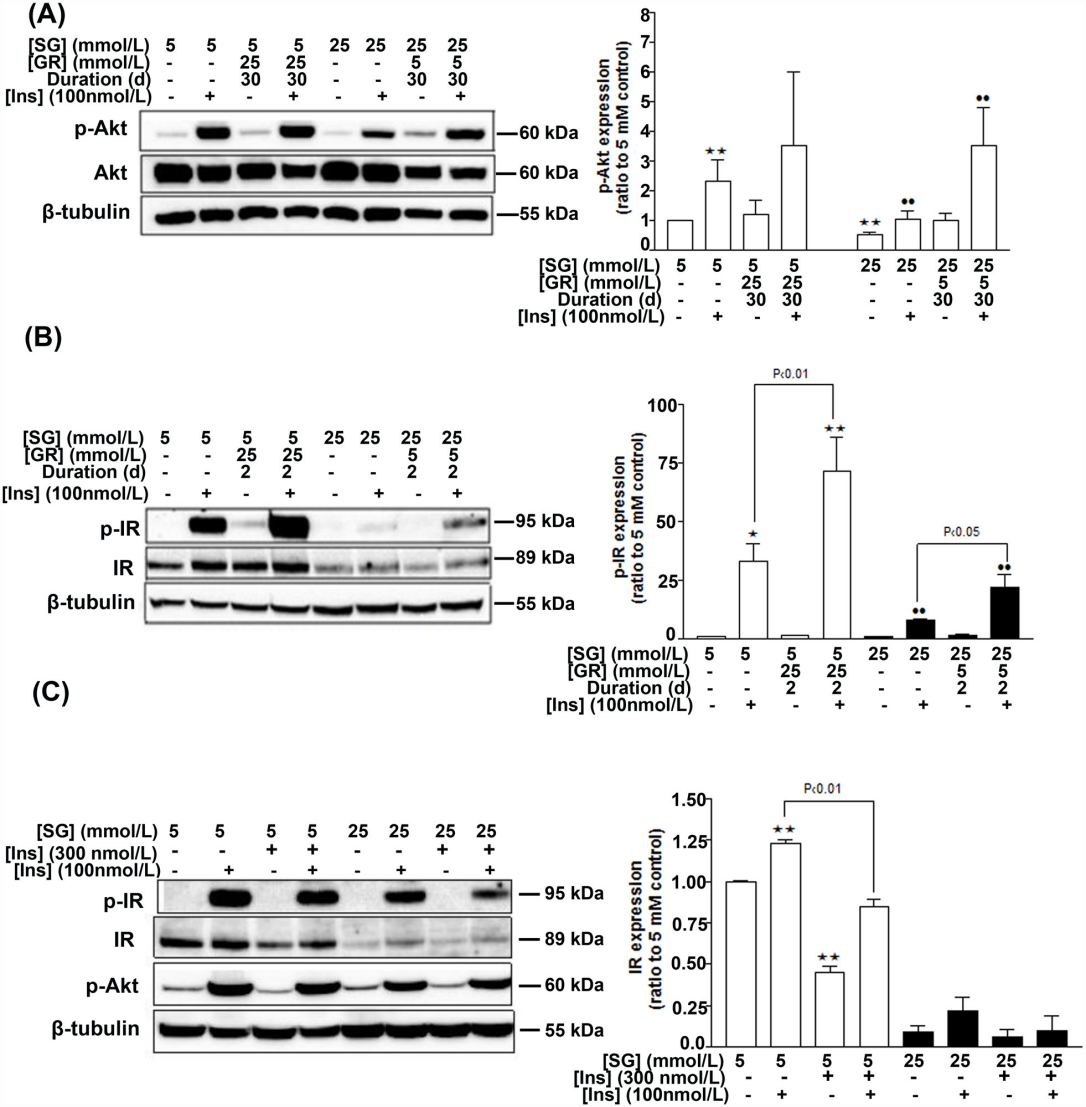
Effects of glucose concentration alterations and prolonged insulin treatment on HGEC. Transition from high to normal glucose for 30 d partially restores p-Akt levels and its response to insulin pulse (A; Western blot and densitometric analysis), while 48 h are enough to partially restore p-IR levels in the presence of insulin (15 min treatment) (B; Western blot and densitometric analysis). Transition from normal to high glucose for 48 h further potentiates IR phosphorylation in response to insulin (B). Prolonged insulin treatment (300 nmol/L Levemir for 24 h) resulted in downregulation of IR levels without affecting its phorsphorylation capacity or p-Akt levels (C; Western blot and densitometric analysis). Densitometric analyses for p-Akt and p-IR are not shown. Data represent mean±SEM, n = 3, *P<0.05, **P<0.01 vs. control (5 mmol/L glucose) and ^••^P<0.01 vs. control (25 mmol/L glucose). SG; starting glucose, GR; glucose replacement.

In order to test whether glucose-induced changes of the insulin signaling pathway were reversible, HGEC permanently cultured in NG were exposed to high glucose concentration for 2–30 days, while HGEC permanently cultured in HG were exposed to normal glucose concentration for the respective time period. We observed that reduced Akt phosphorylation in HG cells was reversible following restoration of glucose levels to normal (NG) for 30 days (both basal and under stimulation by insulin pulse; Fig 3A). Variable p-Akt levels were observed following insulin stimulation of NG-treated cells transferred to HG for 30 days (Fig 3A). Moreover, IR phosphorylation in response to insulin pulse was dependent on glucose concentration. Specifically, a 2-day shift of HG-treated cells to NG or of NG-treated cells to HG, followed by insulin stimulation, was sufficient to up-regulate IR phosphorylation levels by 13% and 38%, respectively (Fig 3B).

In order to simulate conditions of insulin resistance in our *in vitro* model, we incubated HGEC with insulin for 24 h (termed as prolonged insulin treatment) and examined components of the insulin signaling pathway. We observed that prolonged insulin treatment did not affect IR and Akt phosphorylation levels; however, basal IR expression levels were down-regulated in both the NG- and HG- treated groups, both in the presence and absence of insulin pulse (Fig 3C).

### High glucose predisposes HGEC to apoptosis

**Fig 4:**
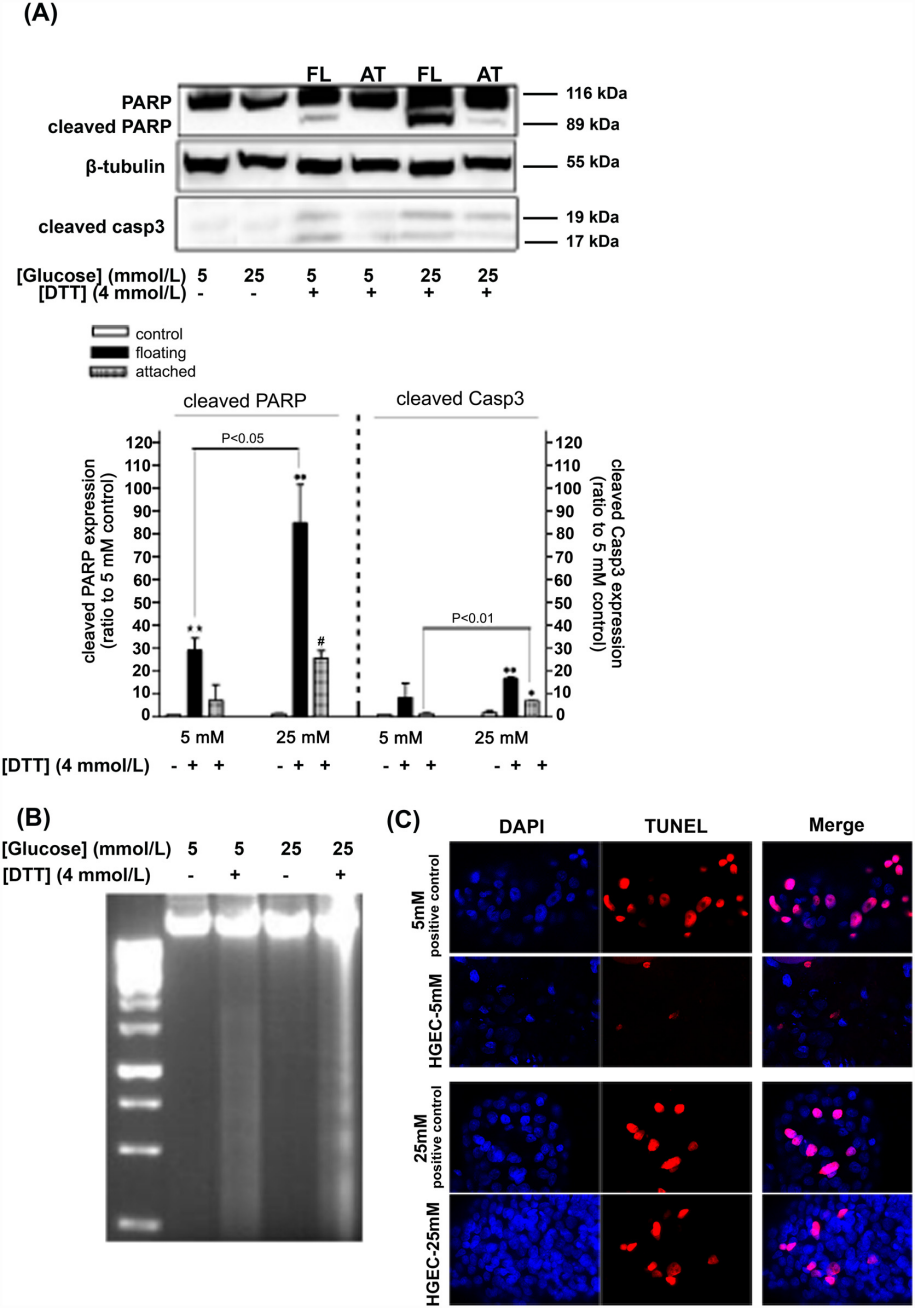
HG predisposes HGEC to apoptosis. Treatment of HGEC with DTT for 24 h results in increased PARP and Caspase-3 cleavage, a response that occurs in a greater extent in adherent 25 mmol/L glucose-culture cells than in control (5 mmol/L glucose-cultured cells) (A; Western blot image and densitometric analysis; FL-floating cells, AT-attached cells). Increased apoptosis is further confirmed by DNA fragmentation analysis of the floating cells under these treatments (B). TUNEL assay shows apoptosis in HG-treated cells, in the absence of DTT, where DAPI (blue) and TUNEL (red) stain nuclei of viable and apoptotic cells, respectively (C). Data represent mean±SEM, n = 3–5, **P<0.01 vs. control (5 mmol/L glucose); ^•^P<0.05, ^••^P<0.01 vs. control (25 mmol/L glucose) and ^#^P<0.05 vs. 5mmol/L-DTT treated cells.

In order to discern differential sensitivity to apoptosis under NG and HG conditions HG- and NG- treated cells were exposed to the apoptotic inducer DTT [37]. Subsequent analysis for apoptotic markers performed in both floating and adherent cells, following DTT treatment (4 mmol/L -24 h), resulted in increased PARP cleavage and Casp3 activation in both floating and adherent HG-treated cells (Fig 4A). In the case of adherent NG-treated cells, DTT did not exert any changes in cleaved PARP and cleaved Casp3 levels (Fig 4A).

Terminal apoptosis, as seen by DNA fragmentation and ladder formation, was evident in floating HG-cultured cells following DTT treatment; NG-treated cells following DTT treatment revealed only a smear (Fig 4B). These data are in agreement with immunoblotting results which indicated PARP cleavage and Casp3 activation in floating HG-treated cells, but not in adherent NG-treated cells. Further confirmation of apoptosis in HG-treated cells, in absence of DTT, was obtained by TUNEL staining, where a greater number of apoptotic nuclei were identified under HG than compared to NG treatment (Fig 4C).

### Effects of high glucose on the insulin signaling pathway in isolated glomeruli

**Fig 5:**
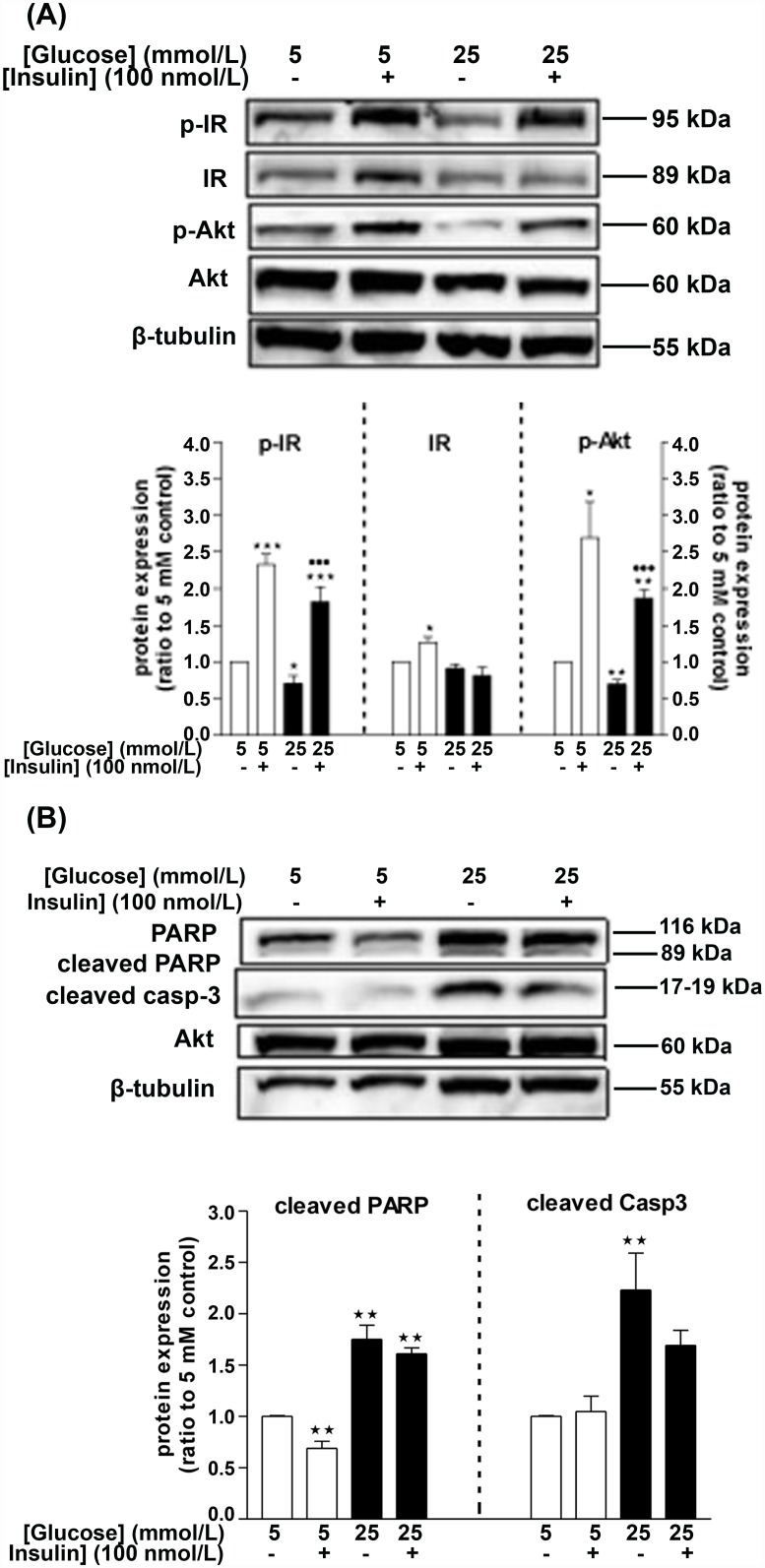
Effects of HG (4d treatment) on the insulin signaling pathway and the survival of isolated glomeruli. Treatment of glomeruli with 25 mmol/L glucose for 96 h resulted in downregulation of the phosphorylated levels of Akt and IR, without affecting total IR levels (A; Western blot image and densitometric analysis), which coincided with enhanced apoptosis as evident by increased PARP and Casp3 cleavage (B; Western blot image and densitometric analysis). Data represent mean±SEM, n = 3–5, *P<0.05, **P<0.01, ***P<0.001 vs. control (5 mmol/L glucose) and ^•••^P<0.001 vs. 25 mmol/L glucose-treated glomeruli.

Having established impairment of insulin signaling in HG-treated HGEC, we investigated activation of this pathway in isolated glomeruli treated with HG for 96 h. HG triggered downregulation of basal levels of both p-IR and p-Akt by 30% (Fig 5A). Interestingly, insulin pulse caused similar increases of Akt and IR phosphorylation in both NG and HG conditions, and also resulted in a small significant increase of IR expression levels in NG-treated glomeruli (Fig 5A). We then examined whether HG predisposed isolated glomeruli to apoptosis. Treatment of glomeruli for 96 h with HG resulted in increased PARP and Caspase-3 cleavage by 75% and 123%, respectively, and insulin pulse experiments resulted in significant reduction of basal PARP cleavage in NG-cultured glomeruli (Fig 5B). These data suggest that HG itself promotes apoptosis in isolated glomeruli.

## Discussion

**Fig 6:**
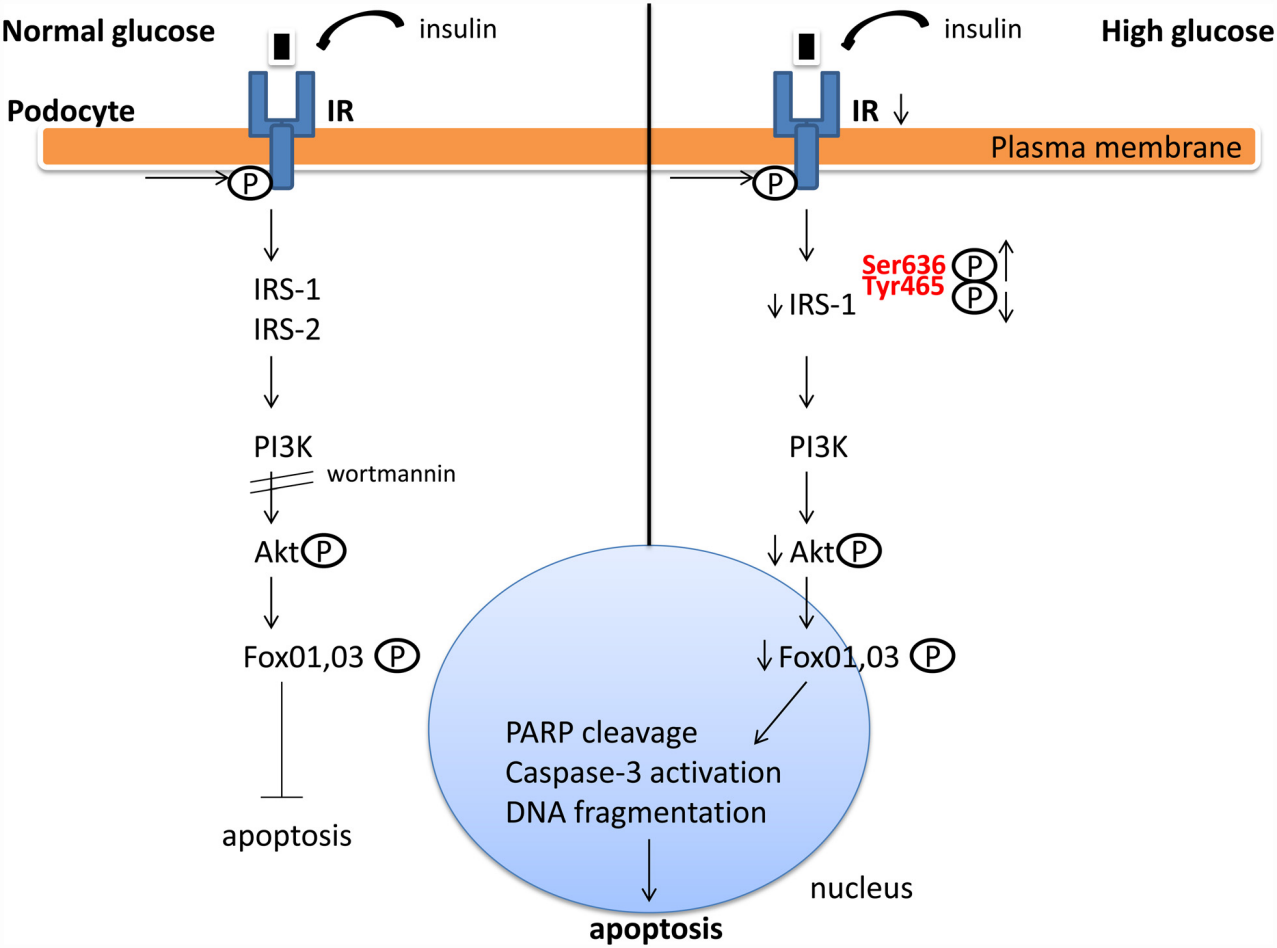
Impaired insulin signaling in the diabetic podocyte results in apoptotic death. HG causes downregulation of IR, IRS-1 levels, increased phosphorylation of IRS-1 at Ser636 and decreased phosphorylation of Akt and Fox01,03a. All these effects synergize to yield an apoptotic outcome. Abbreviations: IR: Insulin Receptor; IRS: Insulin receptor substrate; PI3K: phosphoinositide 3-kinase; Akt: (protein kinase B); PARP: poly (ADP-ribose) polymerase; Fox01,03: forkhead box protein 01, 03.

Hyperglycaemia is a major determinant for the development of diabetic microvascular disease [38] and patients with diabetes experience increased glucose uptake by the kidney [39]. Previous work of our lab has shown that HG induced partial de-differentiation of podocytes [40] and apparently impaired foot processes by severe downregulation of podocalyxin [41]. We presently provided evidence that the presence of HG suffices to downregulate insulin signaling *in vitro* in human podocytes, as well as *ex vivo*, in isolated rat glomeruli. Specifically we demonstrated that under HG conditions in HGEC the pathway was impaired at the level of IR, IRS-1, p-Akt and p-Fox01,03. In the presence of high glucose, IRS1 shifted phosphorylation pattern since it was phosphorylated at Ser636 instead of Tyr465. These findings indicated increased susceptibility to apoptosis [42]. This pathway, which is pivotal for cell survival, was also defective in isolated glomeruli treated with HG: p-IR and p-Akt demonstrated impaired phosphorylation patterns, also suggesting a pro-apoptotic milieu. These results are summarized in Fig 6.

The role of insulin signaling in the development and progression of diabetic nephropathy has been addressed only recently, mostly due to the relatively recent evidence of the critical role of the pathway in normal kidney function [25]. Since then, it was found that there was down-regulation of rat glomerular insulin signaling in diabetes attributed to excessive PKCβ activation [43]. Insofar as the first step of the pathway was concerned, which involves insulin interaction with its receptor, it was demonstrated that IR expression in the kidney is reduced in diabetic human and rat tubular regions [44]. Our study documented that HG suffices to down-regulate IR expression in immortalized human podocytes. The decrease of IR phosphorylation in response to HG could result from either structural/functional inability of the receptor to become phosphorylated in response to insulin, or simply reflect diminished (reduced) IR basal levels. In contrast, in isolated glomeruli, our data indicated that IR phosphorylation in response to HG was reduced but the expression of IR was not altered. Apparently the *ex vivo* experiments are closer to the *in vivo* diabetic conditions, since whole glomeruli were used; these demonstrated that even in the presence of unperturbed levels of IR, HG impaired the process of IR phosphorylation, which is pivotal for insulin survival signaling.

In experiments in which glucose levels were switched from HG to NG, impaired IR phosphorylation was reverted to normal only partly. Moreover, IR phosphorylation was transiently increased in NG-cultured cells transferred to HG, whereas total IR levels remain unchanged; hence this process of “switching” from HG to NG may impair the ability of the receptor to readily adopt to different glucose concentrations. It should be noted that glucose itself has been shown to induce IR tyrosine phosphorylation (which facilitates survival signaling) in pancreatic beta cells [45]; moreover it induced IR serine phosphorylation (pro-apoptotic) at sites close to the C-terminus, via a PKC-mediated mechanism [46]. Serine phosphorylation of the IR can impair subsequent tyrosine phosphorylation and therefore is inhibitory for signal transduction [47,48]. The mechanism by which increased glucose levels induced Ser phosphorylation of IR in podocytes remains to be substantiated. It is possible that “toxic” glucose effect, possibly including conditions of oxidative stress [49], among other factors resulted in phosphorylation of Ser-636, instead of Tyr-465, possibly indicating oxidative cell damage.

In our experiments, prolonged treatment of HGEC with insulin did not result in reduced phosphorylation of the IR, a finding which was observed in adipocytes [50]; instead, the expression of IR was substantially decreased in our system of cultured podocytes. Tiwari and colleagues reported reduced expression of the insulin receptor in the kidneys of insulin-resistant rats [26]; our findings add another parameter leading to decreased IR when compared to “insulin resistance”, the presence of increased insulin levels.

Downstream of the IR, impairment of activation of IRS proteins may play an important role in the development of insulin resistance, or response to increased glucose concentrations. Mice that lack IRS1 display growth retardation and peripheral insulin resistance and [51], while IRS1 and IRS2 deficiency was linked to PI3K inactivation and abolishment of insulin survival responses in the liver and heart of mice [52–54]. Interestingly, IRS-1 protein levels were decreased in the glomeruli of diabetic rats and in glomerular endothelial cells exposed to HG [43]. In our study, we also demonstrated a significant decrease of IRS1 levels in HGEC /podocytes, the unperturbed function of which is pivotal for preservation of selectivity properties of the glomerular filtration barrier. Concomitantly with the HG-induced decrease of IRS-1 protein levels in HGEC, we noted increased Ser636 phosphorylation and reduced Tyr465 phosphorylation of the existing levels of IRS1; suggesting that the activation response of IRS-1 Tyr465 phosphorylation is diminished in HG-treated cells. Similarly to IR, Ser phosphorylation of IRS resulted in impaired insulin signaling; hence it can cause dissociation of IRS from the IR, decreased activation (by reducing tyrosine phosphorylation) or increased susceptibility of IRS to degradation [55,56]. Increased Ser636 and reduced Tyr465 phosphorylation of IRS-1 has been linked to obesity-linked insulin resistance and type 2 diabetes [57–59].

The pro-survival effects of insulin are mediated by Akt phosphorylation and subsequent Fox0 phosphorylation. This step of the pathway is also implicated in the pathogenesis of diabetic nephropathy, since stimulation of Akt phosphorylation was recently shown to improve diabetic nephropathy in mice [60], while renal Akt activity is increased in obese Zucker rats [23]. In addition to these data, our *in vitro* and *ex vivo* models provide evidence that HG treatment of HGEC and glomeruli induces reduction in the phosphorylation levels of Akt, which was partially reversible in the case of podocytes. In the case of HGEC, this suggests that the compromised insulin effect on p-Akt under HG conditions is possibly due to the upstream phosphorylation signal of IRS-1. In support of our findings, HG-mediated downregulation of p-Akt levels has been demonstrated in glomerular endothelial cells [43] and mouse podocytes [61].

Fox0 phosphorylation induces its translocation from the nucleus to the cytoplasm, where it becomes inactivated [62]. It has been demonstrated that insulin action on Fox0 phosphorylation is IRS-dependent and that inhibition of Fox0 phosphorylation results in the development of hyperglycemia, hyperinsulinemia and insulin resistance [52,53]. Reduced Fox01,03 phosphorylation has been shown to correlate with apoptosis in renal mesangial cells [63,64]. Our results demonstrate that HG induced impairment of Fox01,03a phosphorylation possibly as a result of compromised Akt phosphorylation/activation, thus leading to increased apoptosis which was observed in HG-treated podocytes. This suggests that the insulin-mediated prosurvival pathway is also compromised insofar as Fox01,03a inactivation is concerned. The use of insulin pulse protected against apoptosis by increasing Fox01,03a phosphorylation in our cell culture system.

In addition to increased Fox01,03a activation under HG in HGEC, we also obtained direct evidence of induction of apoptosis in HG-treated cells and glomeruli. Specifically, we observed increased PARP and Casp3 activation in HG-treated podocytes following DTT treatment, as well as increased DNA fragmentation and increased TUNEL staining. These data are in agreement to the increased apoptosis observed as a result of HG in mouse podocytes, where it caused increased DNA fragmentation and Caspase-3 activation [61]. In HG-treated glomeruli, we noted increased PARP and Casp3 activation also indicating increased susceptibility to apoptosis. DTT is commonly used for inducing ER stress [65] and therefore, increased sensitivity of HG-treated HGEC to DTT-induced apoptosis may also implicate ER stress in the pathway.

## Conclusions

Overall, this study demonstrates that HG compromises the insulin survival pathway in immortalized human podocytes and rat glomeruli and that this impairment leads to increased susceptibility to apoptosis. Further understanding of the pathobiology of diabetic nephropathy at the level of IR, as well as attempts to revert the glomerular proapoptotic environment attributed to HG, could constitute targets for clinical investigation and therapeutic intervention.

## Abbreviations

HGEC: human glomerular epithelial cells (podocytes)
HG: high glucose
NG: normal glucose
DTT: dithiothreitol
PI3K: phosphoinositide 3-kinase
Akt: (protein kinase B)
IR: insulin receptor
IRS: insulin receptor substrate
Caspase 3: cysteine-dependent aspartate-directed protease caspase 3
PARP: poly (ADP-ribose) polymerase
Fox01,03: forkhead box protein 01, 03
TUNEL: terminal deoxynucleotidyl transferase dUTP nick end labeling
